# Resource‐allocation tradeoffs in caddisflies facing multiple stressors

**DOI:** 10.1002/ece3.3094

**Published:** 2017-06-02

**Authors:** Francisco Correa‐Araneda, Ana Basaguren, Roberto T. Abdala‐Díaz, Alan Mosele Tonin, Luz Boyero

**Affiliations:** ^1^ Laboratory of Ecotoxicology Department of Zoology Faculty of Natural and Oceanographic Sciences University of Concepción Concepción Chile; ^2^ Department of Plant Biology and Ecology Faculty of Science and Technology University of the Basque Country (UPV/EHU) Leioa Spain; ^3^ Department of Ecology Faculty of Science University of Málaga Málaga Spain; ^4^ Department of Ecology IB Universidade de Brasília Brasília, Distrito Federal Brazil; ^5^ IKERBASQUE Basque Foundation for Science Bilbao Spain; ^6^ College of Science and Engineering James Cook University Townsville QLD Australia

**Keywords:** antipredatory defense, larval fitness, resource quality, temperature, Trichoptera

## Abstract

The replacement of native forests by exotic tree monocultures, such as those of *Eucalyptus*, decreases the quality of leaf litter inputs to streams and often reduces riparian cover, which can elevate water temperature. The combined effects of these stressors on the survival and performance of detritivores may be important, as detritivore species loss leads to reduced litter breakdown, a key ecosystem process. Potential loss of cased caddisfly larvae is of particular concern because they are the predominant detritivores in many streams, they are sensitive to warming, and they expend energy on building and carrying their cases, which may be an added burden under times of stress. In a microcosm experiment, we tested whether (i) poor‐quality *Eucalyptus globulus* litter impaired case construction by larvae of *Sericostoma pyrenaicum* (due to preferential allocation of the scarcer available energy to larval fitness) compared to high‐quality *Alnus glutinosa* litter; (ii) whether this effect was enhanced by higher temperatures (15 vs. 10°C) resulting in faster metabolism and greater energy expenditure; but (iii) reduced in the presence of chemical cues from a predatory fish (due to greater investment in more protective cases). We found that *Eucalyptus* had lethal and sublethal effects on larval caddisflies, increasing mortality, reducing growth, and impairing case construction, compared to larvae fed *Alnus*. Temperature did not reinforce the effects of exotic litter on case construction, but predator chemical cues triggered the construction of more protective cases (i.e., longer and better cemented) despite the lower resource quality, providing evidence for environmentally mediated resource‐allocation tradeoffs.

## 1 INTRODUCTION

Many inland waters are subject to multiple anthropogenic stressors (Dudgeon, 2010), which negatively affect biodiversity and cause far‐reaching impacts on ecosystems (Hooper et al., 2012; Vörösmarty et al., 2010). Importantly, co‐occurring stressors act through complex interactions, and often cause unpredictable effects compared to what would be expected from each of those stressors alone (Townsend, Uhlmann, & Matthaei, 2008). Understanding such interactions is particularly relevant when a single anthropogenic alteration leads to the emergence of more than one stressor, because these stressors will tend to occur simultaneously. For example, the replacement of native forests by exotic tree monocultures, a global phenomenon of major concern (FAO 2010) that often affects riparian corridors (Graça, Pozo, Canhoto, & Elosegi, 2002), has two main impacts on stream ecosystems: (i) the quality of leaf litter inputs is generally reduced (Pozo, González, Díez, Molinero, & Elosegi, 1997), and (ii) water temperature can increase in relation to the reduced riparian cover (Bärlocher & Graça, 2002; Ferreira, Elosegi, Gulis, Pozo, & Graça, 2006).

Reduced litter quality and rising temperature are known to have synergistic effects on the performance of some organisms that are key processors of leaf litter in streams (i.e., leaf‐feeding detritivores) (Correa‐Araneda et al., 2015; Ferreira, GonçAlves, Godbold, & Canhoto, 2010). In these ecosystems, where food webs often are detritus‐based (Wallace, Eggert, Meyer, & Webster, 1997), the impacts of environmental changes on the survival and performance of detritivores are very important because the loss of detritivore species directly impacts key ecosystem processes (Gessner et al., 2010). Of particular concern are larvae of some insect groups such as caddisflies (Trichoptera), because (i) they generally are the dominant leaf‐feeding detritivores in streams (Boyero et al., 2011a); (ii) they are particularly sensitive to warming, being evolutionarily adapted to cool waters (Boyero et al., 2012); and (iii) their energetic requirements are high because they construct and carry a portable case, which offers protection from predators (Otto & Svenson, 1980) but involves substantial energy expenditure (Otto, 1974).

Organisms need to allocate the available energetic resources to different traits or functions such as growth, reproduction, and defense (Levins, 1968). Given that resources are generally finite, their allocation to one trait will be at the expense of other traits, resulting in tradeoffs that often depend on multiple factors (Stearns, 1992). In cased caddisfly larvae, the energy expenditure in case building can affect the adult reproductive success or its flying ability (Stevens, Hansell, Freel, & Monaghan, 1999). It is thus plausible that a reduction in litter nutritional quality will modify their tradeoff in resource allocation, favoring growth and nutrient reserves (hence larval development and fitness and, ultimately, adult fitness; Jannot, Bruneau, & Wissinger, 2007) over case building (therefore reducing protection from predators; Nislow & Molles, 1993). This effect is likely to be exacerbated at higher temperatures, which increase metabolic rates and energetic requirements (Brown, Gillooly, Allen, Savage, & West, 2004). However, building a larger or tougher case might be critical in situations where predation risk is perceived (Boyero, 2011; Boyero, Rincón, & Bosch, 2005; Wissinger, Whissel, Eldermire, & Brown, 2006), so the presence of a predator might elicit greater energy expenditure in case building even when resources are limited, curtailing larval development (Mondy, Cathalan, Hemmer, & Voituron, 2011).

**Figure 1:**
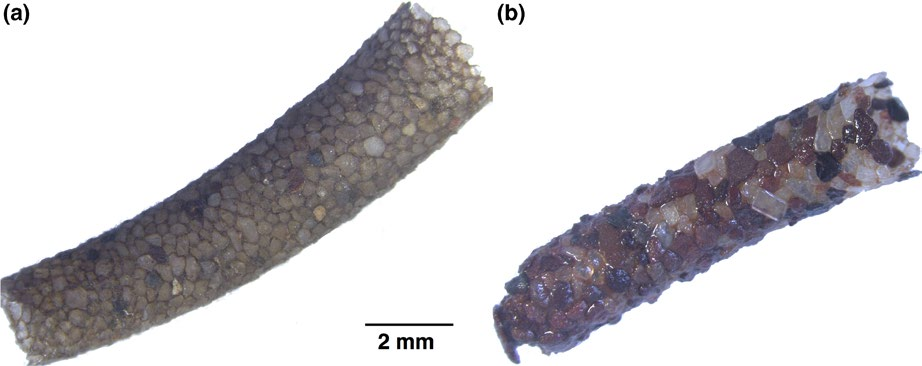
Cases of *Sericostoma pyrenaicum* larvae (a) collected from the field and (b) rebuilt during the experiment

**Figure 2:**
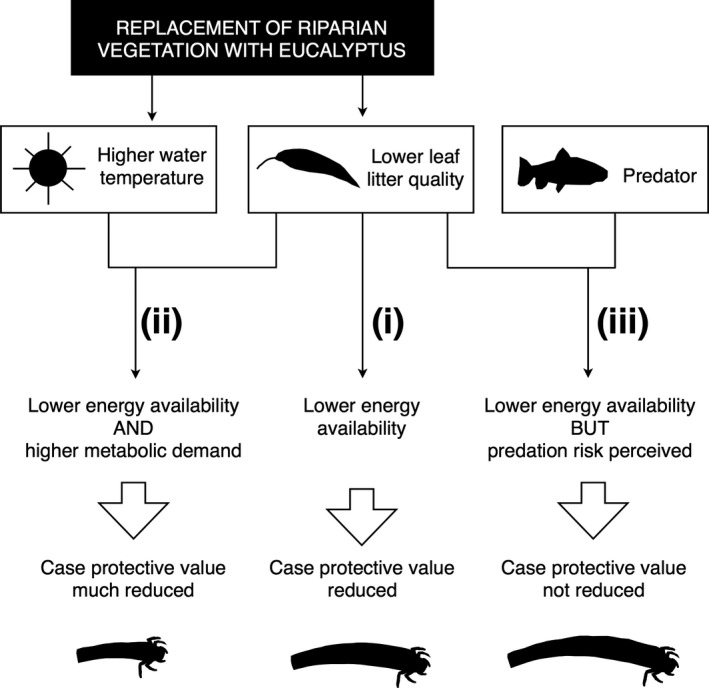
Graphic representation of our predictions that (i) poor‐quality *Eucalyptus* leaf litter would impair case construction, and (ii) the effect would be greater at higher temperatures (due to greater metabolic demand), but (iii) lower in the presence of predators (which would lead to investment in more protective cases)

We tested the above predictions in experimental microcosms using larvae of the caddisfly *Sericostoma pyrenaicum* Pictet, 1865 (Sericostomatidae), a common detritivore in headwater streams in northern Spain that builds a cylindrical, curved case out of fine mineral particles (Figure 1a; Delgado & Carbonell, 1997). This species seems to occupy the ecological niche of *Sericostoma personatum* Spence, which is widely distributed across Europe, as reported for *Sericostoma vittatum* Rambur in other regions of the Iberian peninsula (Feio & Graça, 2000). As many other regions of the world, large areas of the Iberian peninsula have suffered the replacement of native forests with monocultures of *Eucalyptus globulus* Labill., which in many cases have also replaced riparian vegetation and now provide the main litter inputs to streams (Pozo et al., 1997). We provided caddisflies with leaf litter of *E. globulus* and, for comparison, *Alnus glutinosa* (L.) Gaertn, the most common and widespread native riparian tree in the region, to assess the effects of poor‐ vs. high‐quality litter on caddisfly resource allocation to larval fitness (i.e., growth and/or nutrient reserves) or case building. Higher temperatures have been reported in streams flowing through *Eucalyptus* plantations than in streams flowing through native deciduous forests (Ferreira et al., 2006), so we kept caddisflies at one of two temperatures (10°C or 15°C) to examine the effects of temperature on resource allocation. Although this was a substantial difference, both temperatures were within the range commonly experienced by *S. pyrenaicum* in the region during the autumn, when the study was conducted (Martínez et al., 2016; Pozo et al., 2011). Finally, we examined the effects of predators by adding water‐borne chemical cues from a native predatory fish (*Salmo trutta* L.) to some caddisflies but not others. We hypothesized that (i) poor‐quality *E. globulus* litter would impair case construction, as larvae would preferentially allocate the scarcer available energy to improve larval fitness; (ii) this effect would be greater at higher temperature (due to faster metabolism, hence greater energy expenditure); but (iii) the effect would be lower in the presence of predators (which would lead to investment in more protective cases) (Figure 2).

## 2 MATERIALS AND METHODS

### 2.1 Organism collection

We collected the leaf litter, caddisflies, and fish to be used in the experiment in the Agüera catchment in northern Spain (43°N, 3°W). Freshly abscised leaves of *A. glutinosa* (hereafter *Alnus*) and *E. globulus* (hereafter *Eucalyptus*) were collected from the riparian forest floor, transported to the laboratory and air‐dried to constant mass. Larvae of *S. pyrenaicum* were collected from natural litter packs using 250‐μm‐mesh hand nets and transported to the laboratory in aerated stream water. Two juvenile individuals of the brown trout, *Salmo trutta* (Linnaeus, 1758) from the Agüera stream were obtained from a fish breeding center at Trucíos (Diputación Foral de Bizkaia) and transported to the laboratory in an aerated aquarium. Caddisflies were starved for 24 hr before the experiment and trout were starved for the duration of the experiment.

### 2.2 Experimental procedure

The experiment was undertaken in microcosms, which consisted of glass jars containing a mixture (50:50) of dechlorinated tap water and filtered (50 μm) stream water (350 ml), sand collected from the stream substrate and incinerated (600°C, 4 hr), and leaf litter (0.4 g ± 0.006 SD dry mass) of either *Eucalyptus* or *Alnus* that had been previously leached for 24 hr in separate jars. Microcosms were provided with aeration and placed within a water bath at either 10°C or 15°C. Caddisfly larvae were carefully pushed out of their cases using soft entomological forceps and introduced in the experimental microcosms. Once daily, microcosms received 5 ml of water from the trout aquarium (thus containing water‐borne cues from the predatory fish) or water from another aquarium containing the same mixture of stream and tap dechlorinated water but no fish. All combinations of litter, temperature, and predator were replicated ten times. Additionally, there were five control microcosms (with no caddisflies) for each litter and temperature combination, which were used to estimate litter mass loss due to microbial processing. The experiment continued for seven days, the necessary time for caddisflies to build cases of similar size to original cases, during which a natural photoperiod of 12:12 hr of light:dark was maintained.

### 2.3 Chemical and physical analysis

Caddisfly original cases were dried and weighed to the nearest 0.0001 g and photographed on a Leica stereoscope. Pictures were processed through the software Leica Application Suite 4.0 (LAS; Leica Microsystems) in order to calculate their maximum length and their front and rear widths (as front width was larger than rear width) with a precision of 0.01 mm. At the end of the experiment, caddisflies were gently pushed out of their rebuilt cases, which were lyophilized, weighed, and photographed to calculate length and width as above. We determined case toughness using a penetrometer, which measured the grams of force required to pierce the case with a 1.55‐mm‐diameter steel rod. Finally, cases were incinerated (600°C, 4 hr) and reweighed to estimate their proportion of organic and inorganic materials.

The initial caddisfly body mass (BM, in mg) was estimated from the initial case front width (CW, in mm), based on the following relationship derived from 20 extra‐individuals: BM = −0.001716 + (0.001334 × CW). This relationship provided a better fit (*r* ^2^ = .50, *p* < .0005) than the relationship of body mass with case length or the rear case width. At the end of the experiment, caddisflies were lyophilized and weighed. Their carbon (C) and nitrogen (N) concentrations (% of total dry mass) were determined using a CNH Perkin‐Elmer 2400 elemental analyzer (Perkin Elmer, Norwalk, Connecticut). Initial litter quality was examined based on the concentration of C and N (as above), phosphorus (P, measured spectrophotometrically after autoclave‐assisted extraction; APHA 1998), and phenolic compounds (determined using phloroglucinol on homogenized and centrifuged samples; Abdala‐Díaz, Cabello‐Pasini, Márquez‐Garrido, & Figueroa, 2014), using five replicates in each case. At the end of the experiment, leaves were oven‐dried (60°C, 48 hr) and weighed.

### 2.4 Data analysis

We measured several response variables in each microcosm to examine caddisfly resource allocation to different functions (i.e., growth, nutrient reserves, and case protective value). Caddisfly growth was quantified in two ways: (i) growth relative to initial body mass, calculated as the difference between final and initial body mass (mg) divided by initial body mass (mg); and (ii) growth efficiency, calculated as the efficiency of conversion of ingested food [i.e., the difference between final and initial body mass (mg) divided by the difference between initial and final litter mass in the microcosm (mg)]. Nutrient reserves were quantified as the N concentration (i.e., proportion of total body mass). The case protective value was quantified using three metrics: the relative organic content (i.e., the proportion of total case mass made of organic material), case length (mm), and case toughness (g). Finally, we recorded whether each individual was dead or alive at the end of the experiment to examine any potential effects of litter type, temperature, or predator presence on survival.

We used linear models [gls function and restricted maximum likelihood (REML) method, “nlme” package (Pinheiro, Bates, DebRoy, & Sarkar, 2016), R software v. 3.3.1 (R Core Team 2016)] to test effects of litter type (1st hypothesis), temperature, predator presence, and their interactions, including litter type × temperature (2nd hypothesis) and litter type × predator presence (3rd hypothesis), on larval growth, growth efficiency, and N content and case organic content, length, and toughness. For these analyses, we (1) removed data from microcosms with dead caddisflies (17 microcosms); (2) searched for and removed potential outliers using Cleveland dot‐ and boxplots; (3) used multipanel boxplots to assess whether variances were homogeneous, which required the use of a variance structure [VarIdent function (Pinheiro et al., 2016)]; (4) used a backward model selection procedure based on AIC to select the best fit model (Table S1); and (5) explored residuals to ensure that there were no visual patterns, and that linear model assumptions were not violated (Zuur & Ieno, 2015; Zuur, Ieno, Walker, Saveliev, & Smith, 2009). We used a second set of models including initial caddisfly body mass as a covariate because, despite random assignment of individuals to treatments, we detected a statistically significant difference between temperature treatments (*F*_1,62_ = 6.52, *p* = .0132); as results remained the same, we kept the first set of models. When a litter type × temperature or litter type × predator interaction was significant, we fitted separate models for each litter type to examine temperature and/or predator effects. Effects of litter type, temperature, predator presence, and their interactions on larval survival were tested with a binomial generalized linear model [also called logistic regression; glm function and clog‐log identity link (as there were more alive than dead individuals); “stat” R package] based on chi‐square estimates and using a backward model selection procedure (Zuur et al., 2009).

## 3 RESULTS

*Eucalyptus* litter had lower N and P concentrations and N:P ratio and higher C:N ratio than *Alnus* litter, but the concentration of phenolic compounds was similar in both litter types (Table 1). All caddisflies built a case during the experiment, starting within the first 4 hr. Rebuilt cases differed from the original ones in that they were straight, rather than slightly curved (Figure 1b). More caddisflies died when fed *Eucalyptus* litter than when fed *Alnus* litter (14 vs. 3 dead larvae; deviance = 9.65, *df* = 1, 78, *p* = .0019).

**Table 1:** Mean (±standard deviation) of nitrogen (N) and phosphorus (P) concentration (% dry mass), C:N and N:P ratios, and phenolic compound concentration (% dry mass), for *Alnus* and *Eucalyptus* leaf litter based on measurements of five replicates. Different letters indicate significant differences on the basis of linear models (significant values *p* < .05)

Species	N	P	C:N	N:P	Phenolic compounds
*Alnus glutinosa*	2.10 ± 0.12^a^	0.043 ± 0.001^a^	27.98 ± 1.16^b^	121.39 ± 6.69^a^	2.89 ± 1.17
*Eucalyptus globulus*	0.82 ± 0.05^b^	0.038 ± 0.002^b^	78.37 ± 4.85^a^	54.53 ± 3.37^b^	2.19 ± 0.63

**Figure 3:**
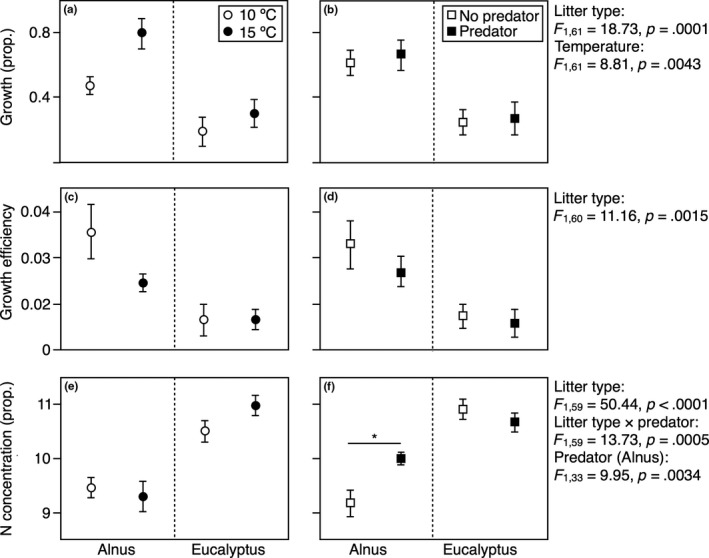
Variation (mean ± standard error) in (a, b) caddisfly larval growth (proportion), (c,d) growth efficiency [growth (mg) relative to consumption (mg)], and (e, f) nitrogen content (proportion) with litter type (native *Alnus glutinosa* vs. exotic *Eucalyptus globulus*) and its interactions with (a, c, e) temperature (10 vs. 15°C) and (b, d, f) the presence of chemical cues from predatory fish. Significant effects (*F* and *p* values resulting from linear models, see text) are indicated on the right‐hand side of graphs

**Figure 4:**
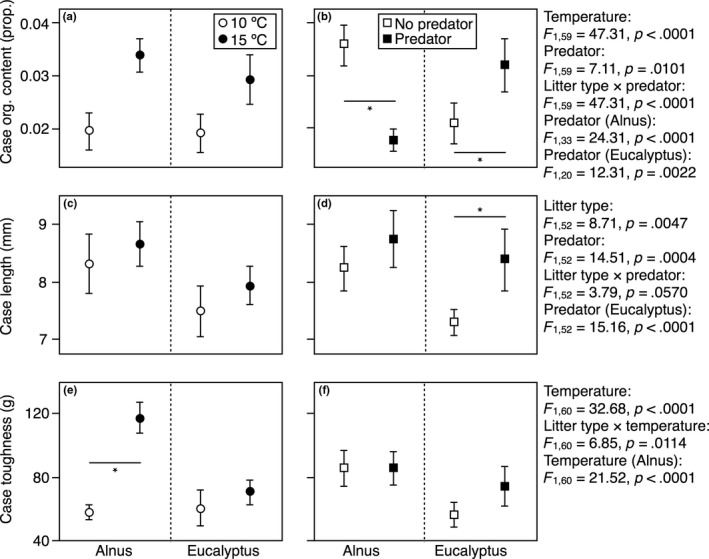
Variation (mean ± standard error) in (a, b) case organic content (proportion), (c, d) case length (mm), and (e, f) case toughness (g necessary to pierce the case using a penetrometer) with litter type and its interactions with (a, c, e) temperature and (b, d, f) the presence of chemical cues from predatory fish (treatments as in Figure 1 legend)

Surviving larvae grew more when fed *Alnus* and at 15°C (Figure 3a), with no effect of predator cues (Figure 3b). Growth efficiency was higher on *Alnus*, with no temperature or predator effects (Figure 3c,d). N concentration was higher on larvae fed *Eucalyptus* (Figure 3e) and, when fed *Alnus*, it was higher with predator cues (significant litter type × predator interaction; Figure 3f). Cases built at 15°C had more organic content than those built at 10°C (Figure 4a); case organic content also varied depending on predator presence, but differed between litter types (significant litter type × predator interaction): when they detected the predator, larvae built cases with higher organic content when fed *Eucalyptus*, and lower organic content when fed *Alnus* (Figure 4b). Case length was greater for larvae fed *Alnus* (Figure 4c), and there was a tendency for greater case length in the presence of predators when larvae were fed *Eucalyptus* (*p* = .057); when litter types were examined separately, larvae fed *Eucalyptus* had larger cases when they detected a predator, while there was no difference for larvae fed *Alnus* (Figure 4d). Case toughness was greater at 15°C for larvae fed *Alnus* (significant litter type × temperature interaction; Figure 4e); an apparent trend for tougher cases in the presence of predators when larvae were fed *Eucalyptus* was not significant (Figure 4f).

## 4 DISCUSSION

Our experiment showed how reduced litter quality and increased temperature, two stressors often co‐occurring in stream ecosystems flowing through exotic plantations (Lynch, Rishel, & Corbett, 1984), can modify resource‐allocation tradeoffs in *Sericostoma* larvae, with potential consequences for larval fitness and vulnerability to predation. Like many other caddisfly larvae, *Sericostoma* larvae build a portable case that offers protection from predators (Otto & Svenson, 1980), but involves important energy expenditure (Otto, 1974). This implies that when resources are scarce, their allocation to fitness or defensive traits may become critical for survival and reproduction (Stearns, 1992; Stevens et al., 1999).

Quality of *Eucalyptus* litter was poorer than *Alnus* litter, because of its lower nutrient concentration, and possibly also because of its higher toxicity. Although we found no differences in the concentration of phenolic compounds in the two species, it is likely that *Eucalyptus* litter had higher concentration of other toxic compounds such as tannins and/or essential oils (Graça et al., 2002). The poorer quality of the exotic litter would explain its lethal and sublethal effects on caddisfly larvae in our experiment: mortality was almost five times greater for larvae fed *Eucalyptus* litter, which also inhibited larval growth and growth efficiency. Other studies have reported reduced growth of caddisfly larvae fed exotic vs. native litter; for example, growth of larval *Anisocentropus kirramus* Neboiss, 1980 was inhibited when they were fed litter of the exotic camphor laurel [*Cinnamomum camphora* (L.) Nees et Eberm.] compared to native litter (Davies & Boulton, 2009). Unexpectedly, larvae fed *Eucalyptus* had higher N concentration in their bodies at the end of the experiment than those fed N‐rich *Alnus* litter; although apparently contradictory, this finding could be due to dilution by gut contents as larvae fed *Alnus* probably had greater amounts of litter in their guts, which would underestimate larval N concentration (Fagan et al., 2002).

As predicted, *Eucalyptus* litter impaired case construction: larvae built longer cases at both temperatures and tougher cases at 15°C when fed *Alnus* than when fed *Eucalyptus*. It is thus likely that larvae preferentially allocated the scarcer energy provided by the exotic litter to improve their fitness, which would be more critical than building a more protective case in the absence of predators. However, as predicted, the tradeoff in resource allocation changed when predation risk was perceived. Caddisflies often select tough materials for case building (Rincón & Martínez, 2006), but this behavior is reinforced when chemical cues from predators are detected (Boyero, 2011; Boyero et al., 2005). Accordingly, when we added chemical cues from trout to the microcosms, larvae fed *Eucalyptus* allocated more time and energy to case building than when there were no chemical cues and built longer cases with higher organic content, implying increased silk production to better cement the case. In contrast, when larvae were fed *Alnus*, the presence of a predator did not affect case length or larval growth and growth efficiency, although it resulted in higher larval N concentration (possibly as a result of higher feeding, and thus dilution by gut contents, in the absence of predators) and produced cases with less organic content (possibly because N was preferentially directed to the body).

Larval growth was higher at 15°C than at 10°C, while larval growth efficiency did not differ between temperatures. Thus, higher growth occurred because larvae consumed more litter at 15°C (linear model, *F*_1,61_ = 7.72, *p *=* *.0072) and probably in relation to higher metabolic rates at higher temperatures (Brown et al., 2004). Larval N concentration did not change with temperature, but cases had more organic content at 15°C, suggesting that temperature favored the allocation of N to silk production. Contrary to our prediction, temperature did not reinforce the effects of reduced exotic litter quality on case organic content or case length: larvae fed *Eucalyptus* built similar cases at both experimental temperatures. Only larvae fed *Alnus* built tougher cases at 15°C, indicating that higher temperatures (within certain limits) may favor the construction of more protective cases, particularly when high‐quality resources are available.

Our findings demonstrate how the replacement of native riparian vegetation with *Eucalyptus* can have important consequences for the survival and fitness of larval caddisfly populations, which play a key role as litter processors in streams. Lethal and sublethal effects of *Eucalyptus* are mediated by the poorer quality and the toxicity of leaf litter entering the stream, which significantly reduce the resources available and can compromise important functions such as larval growth, mostly when predation risk triggers greater energy allocation to case building. The higher temperatures often associated with the logging of riparian vegetation, however, might only be of secondary importance for caddisfly populations (when within normal variation), although they are known to affect key stream ecosystem processes such as litter breakdown (mostly through effects on microbially mediated breakdown rates; Boyero et al., 2011b), and fluxes of energy and biomass between the stream and the riparian ecosystem (Greig et al., 2012). Our results are likely applicable to other detritivorous case‐building caddisflies, which will face similar tradeoffs to those described here when exposed to predators and low‐quality food. Studies using other species will be valuable in order to achieve more general conclusions.

## CONFLICT OF INTEREST

None declared.

## AUTHOR CONTRIBUTIONS

FCA, LB, and AB designed and conducted the experiment. RTAD did the chemical analyses. AMT and LB analyzed the data. LB and FCA wrote the manuscript with feedback from all other authors.
