# Supplementary material for: Resource‐allocation tradeoffs in caddisflies facing multiple stressors
Source: Ecol Evol. 2017 Jun 2;7(14):5103–10. doi: 10.1002/ece3.3094 (PMC5528245; doi:10.1002/ece3.3094)
Supplement: Supplementary file 1 [file ECE3-7-5103-s001.pdf]

Table S1. Summary of backward model selection (using the Akaike information criterion, AIC) for larval growth, growth efficiency and N concentration and case organic content, length and toughness. L, litter type (*Alnus* or *Eucalyptus*); P, predator (present or absent); T, temperature (10 or 15 ºC). P-values refer to the comparison between 1st and 2nd, 2nd and 3rd model, and so on; non-significant p-values ($p > 0.05$) indicate that both models were similar. Different degrees of freedom (df) for each full model is due to the use of different variance structures. Growth, growth efficiency and case organic content were allowed to vary with respect to temperature; N concentration with respect to temperature and predator; and case length and toughness with respect to litter type, predator and temperature;

	Model	df	AIC	p
	**Larval growth**			
**1**	L + P + T + L:P + L:T + P:T + L:P:T	10	46.5	
**2**	L + P + T + L:P + L:T + P:T	9	44.5	0.971
**3**	L + P + T + L:P + L:T	8	42.6	0.737
**4**	L + P + T + L:T	7	40.6	0.971
**5**	L + T + L:T	6	40.2	0.212
**6**	L + T	5	38.4	0.623
	**Larval growth efficiency**			
**1**	L + P + T + L:P + L:T + P:T + L:P:T	10	-257.4	
**2**	L + P + T + L:P + L:T + P:T	9	-259.4	0.815
**3**	L + P + T + L:P + L:T	8	-261.2	0.638
**4**	L + P + T + L:T	7	-262.4	0.379
**5**	L + T + P	6	-262.8	0.205
**6**	L + T	5	-263.8	0.332
**7**	L	4	-263.3	0.108
	**Larval N concentration**			
**1**	L + P + T + L:P + L:T + P:T + L:P:T	16	130.1	
**2**	L + P + T + L:P + L:T + P:T	15	128.9	0.354
**3**	L + P + T + L:P + L:T	14	127.5	0.450
**4**	L + P + T + L:P	13	127.5	0.163
**5**	L + P + L:P	12	125.6	0.762
	**Case organic content**			
**1**	L + P + T + L:P + L:T + P:T + L:P:T	16	-350.2	
**2**	L + P + T + L:P + L:T + P:T	15	-352.2	0.910

**3**	L + P + T + L:P + P:T	14	-353.8	0.548
**4**	L + P + T + L:P	13	-355.1	0.413
	**Case length**			
**1**	L + P + T + L:P + L:T + P:T + L:P:T	16	208.7	
**2**	L + P + T + L:P + L:T + P:T	15	208.9	0.137
**3**	L + P + T + L:P + L:T	14	208.0	0.292
**4**	L + P + T + L:P	13	206.3	0.586
	**Case toughness**			
**1**	L + P + T + L:P + L:T + P:T + L:P:T	16	594.9	
**2**	L + P + T + L:P + L:T + P:T	15	593.0	0.759
**3**	L + P + T + L:T + P:T	14	592.3	0.251
**4**	L + P + T + L:T	13	591.1	0.361
**5**	L + T + L:T	12	590.5	0.242
